# Plasma inflammatory cytokines are associated with cognitive impairment after acute minor ischemic stroke and transient ischemic attack

**DOI:** 10.3389/fimmu.2025.1445938

**Published:** 2025-08-01

**Authors:** PanPan Zhao, Meng Zhao, GuiMei Zhang, WeiJie Zhai, YongChun Wang, YanXin Shen, Li Sun

**Affiliations:** Department of Neurology and Neuroscience Center, The First Hospital of Jilin University, Jilin University, Changchun, China

**Keywords:** post-stroke cognitive impairment (PSCI), acute minor ischemic stroke, transient ischemic attack (TIA), inflammatory cytokines, interleukin (IL)-8, interleukin (IL)-18, matrix metalloproteinase (MMP)-9, macrophage inflammatory protein (MIP)-1α

## Abstract

**Background:**

Inflammation plays a complex role in post-stroke cognitive impairment (PSCI). The purpose of our study was to explore the possible relationship between peripheral blood inflammatory biomarkers and PSCI.

**Methods:**

This prospective observational cohort study included patients with mild ischemic stroke and transient ischemic attack (TIA) who were hospitalized at the First Hospital of Jilin University between April 2019 and March 2022. Fasting plasma, collected on the second day after admission, was used to detect 10 inflammatory cytokines by ELISA and multifactorial assay. The Montreal Cognitive Assessment scale score < 22 was used as the diagnostic criterion for PSCI. We explored the associations between baseline plasma cytokines and PSCI at 0–30 months of follow-up using multivariable logistic regression and further applied structural equation modeling (SEM) to explore the potential pathways.

**Results:**

A total of 236 patients were included in the analysis. Interleukin-18 (IL-18), matrix metalloproteinase-9 (MMP-9), and macrophage inflammatory protein-1α (MIP-1α) were independently associated with PSCI at 3–30 months of follow-up, while IL-18, MMP-9, and IL-8 were independently associated with delayed-onset PSCI (>6 months of follow-up). Similar findings were observed in the sensitivity analysis after excluding the patients with ischemic symptoms with an onset of more than 1 week and those with TIA. The associations of IL-18, MMP-9, and MIP-1α with PSCI remained consistent across age and sex subgroups. In delayed-onset PSCI, IL-18, and MMP-9 showed stable associations, while IL-8 mainly affected patients younger than 65 years. Furthermore, SEM suggested that peripheral inflammation involving MMP-9 and IL-18 mediated the development of PSCI.

**Conclusions:**

The plasma inflammatory markers centered on neutrophils—MMP-9 and IL-18—interact with various risk factors (age, gender, DMTS, DWMH, cerebral atrophy) and contribute to the development of PSCI.

**Clinical Trial Registration:**

https://www.chictr.org.cn/, identifier ChiCTR1900022675.

## 1 Introduction

Post-stroke cognitive impairment (PSCI) refers to cognitive dysfunction after a stroke caused by any cause (1). The survival rate of ischemic stroke has increased, and the disability rate has decreased due to advances in intravenous thrombolysis and intravascular therapy for acute ischemic stroke (2–5). In contrast, the incidence of cognitive impairment after ischemic stroke shows an upward trend. In patients with minor stroke and transient ischemic attack (TIA), the PSCI incidence ranges from 4.4% to 77% (1, 2, 6, 7), directly affecting the quality of life (1). However, there is no specific treatment for PSCI because the underlying etiology remains unclear.

Both acute ischemic events (1) and chronic neurodegeneration (2, 8) are involved in the development of PSCI, with inflammatory immunity also playing a significant role (9, 10). Previous studies have investigated the associations between interleukin-6 (IL-6), IL-8, IL-12, IL-1β, IL-10, C-reactive protein, and PSCI (9–11). However, clinical findings on inflammatory immunity and PSCI remain controversial (10), and studies on IL-18, matrix metalloproteinase-9 (MMP-9), and PSCI are still limited. Structural equation modeling (SEM) is a technique used to elucidate complex relationships among variables by incorporating causal assumptions into multiple regression models. This technique allows one variable to be both an “exposure” on one path and an “outcome” on another, and it can quantify both direct and indirect effects of one variable on another. Therefore, SEM efficiently reveals the complex relationships between dependent and independent variables. We will use the SEM to further explore the association between baseline plasma inflammatory cytokines and cognitive function at baseline and during follow-up, based on a longitudinal cohort study of cognition after acute minor ischemic stroke and TIA.

## 2 Methods

### 2.1 Study populations

The population in this study included mainly patients from Clinical Investigation for Vascular Cognitive Impairment in Ischemic Cerebrovascular (CI-VCI-IS), which was registered with the Chinese Clinical Trial Registry (URL: https://www.chictr.org.cn/; unique identifier: ChiCTR1900022675). The CI-VCI-IS study is one single-center prospective cohort study in Northeast China, in which patients with acute mild stroke and TIA admitted to the neurology ward of the First Hospital of Jilin University from April 2019 to March 2022 were continuously enrolled, with follow-ups conducted every 3 months. The inclusion criteria primarily involved patients diagnosed with cerebral ischemia aged 50–80 years. The exclusion criteria were cognitive impairment from any cause before this disease and those who were unable to cooperate with the screening scale caused by visual impairment, deafness, aphasia, and so on. The following inclusion criteria were applied in this analysis (1): a diagnosis of TIA or acute cerebral infarction meeting the World Health Organization criteria (12) within 2 weeks of onset (2), a National Institutes of Health Stroke Scale (NIHSS) score of ≤6 (13), and (3) Hematological specimens were collected at baseline.

### 2.2 Ethics approval

The research was carried out following the World Medical Association Declaration of Helsinki and was approved by the First Hospital of Jilin University Ethics Committee (protocol code 19K023-003). All procedures conformed to national and institutional guidelines, and written informed consent was obtained from all participants or their relatives.

### 2.3 Study variables

In line with previous studies on PSCI, we collected demographic variables such as age, gender, years of education, history of previous stroke, and hypertension. Serum peripheral inflammation indicators included neutrophil count and cytokines. Imaging indicators included white matter hyperintensities, stroke characteristic variables, diameter of maximum transverse section (DMTS), and NIHSS score at admission.

### 2.4 Monitoring of cytokines

Plasma samples were obtained from the Department of Biobank, Division of Clinical Research, First Hospital of Jilin University, which was separated by centrifugation at 2000 rpm for 15 min and then stored in Eppendorf tubes at −80°C, using fasting blood samples collected after admission. Subsequently, the concentrations of cytokines—tumor necrosis factor-α (TNF-α), MMP-9, IL-8, monocyte chemoattractant protein-1 (MCP-1), macrophage inflammatory protein-1α (MIP-1α), intercellular adhesion molecule-1 (ICAM), brain-derived neurotrophic factor (BDNF), and IL-18—were measured using a multiplex assay, following the instructions provided in the Luminex manual (R&D Systems, LXSAHM-08, USA). In contrast, the concentrations of myeloperoxidase (MPO) and high mobility group box 1 (HMGB1) were measured using the double-antibody sandwich ELISA technique, according to the Human MPO ELISA Kit manual (Proteintech Group, Inc.) and the Human HMGB1 ELISA Kit manual (Elabscience Biotechnology Co., Ltd.).

### 2.5 Assessment of cognitive function and outcome measurement

We performed a Mini-Mental State Examination (MMSE) and Montreal Cognitive Assessment (MoCA) for overall cognition. All baseline scales were assessed 7–10 days after the patient’s ischemic event, followed by assessments every 3 months thereafter. Professional evaluators performed these assessments with over 5 years of experience. For patients with less than 12 years of education, 1 point was added to the total MMSE/MoCA score. A MoCA score of <22 was used as the basis for whether there is PSCI (14). For patients followed up within 2.5 years who had multiple follow-up visits, the assessment conducted within 6–12 months was the first choice as the outcome variable. Furthermore, patients who developed cognitive impairment during the 3- to 6-month follow-up were classified as having early onset PSCI (ePSCI), while those who developed impairment after 6 months were classified as having delayed-onset PSCI (dPSCI) (15, 16).

### 2.6 Statistical analysis

Measurement data were expressed as the median and interquartile range (IQR), and counting data were presented as frequency and percentage. First, due to the large-scale variation of the multi-factors, Z-score normalization was performed to convert the original data into a standard normal distribution with a mean of 0 and a standard deviation of 1. That is, Z = (raw data value − mean of the raw data)/standard deviation (SD). The relationship between each cytokine and baseline, early onset and delayed-onset, and total follow-up cognitive function was analyzed using univariate logistic regression analysis. Then, confounding factors were adjusted for age, sex, and years of education. In addition, adjustments were made for previous stroke, hypertension, NIHSS scores, deep white matter hyperintensity (DWMH), DMTS, Fezakas scores, cerebral atrophy, and the number of intracranial vascular stenosis. Second, further sensitivity analysis was performed by excluding patients with ischemic symptom onset beyond 1 week and additionally excluding those with TIA. Then, subgroup analyses were performed based on sex and age (≥65 years), and interaction effects were evaluated to quantify consistency across subgroups. Finally, correlation analysis was conducted, and the SEM was developed based on previous research and correlation results. The model was adjusted and refined according to fit assessment indicators, recommended improvement, and previous literature. The goal was to optimize model fit indices while ensuring clinical relevance. The statistical analyses were performed using Stata software version 15.0 (StataCorp, College Station, TX, USA) and R software version 4.3.2 (http://www.r-project.org/). A two-tailed *p*-value < 0.05 was considered statistically significant.

## 3 Results

### 3.1 Population characteristics

**Figure 1:**
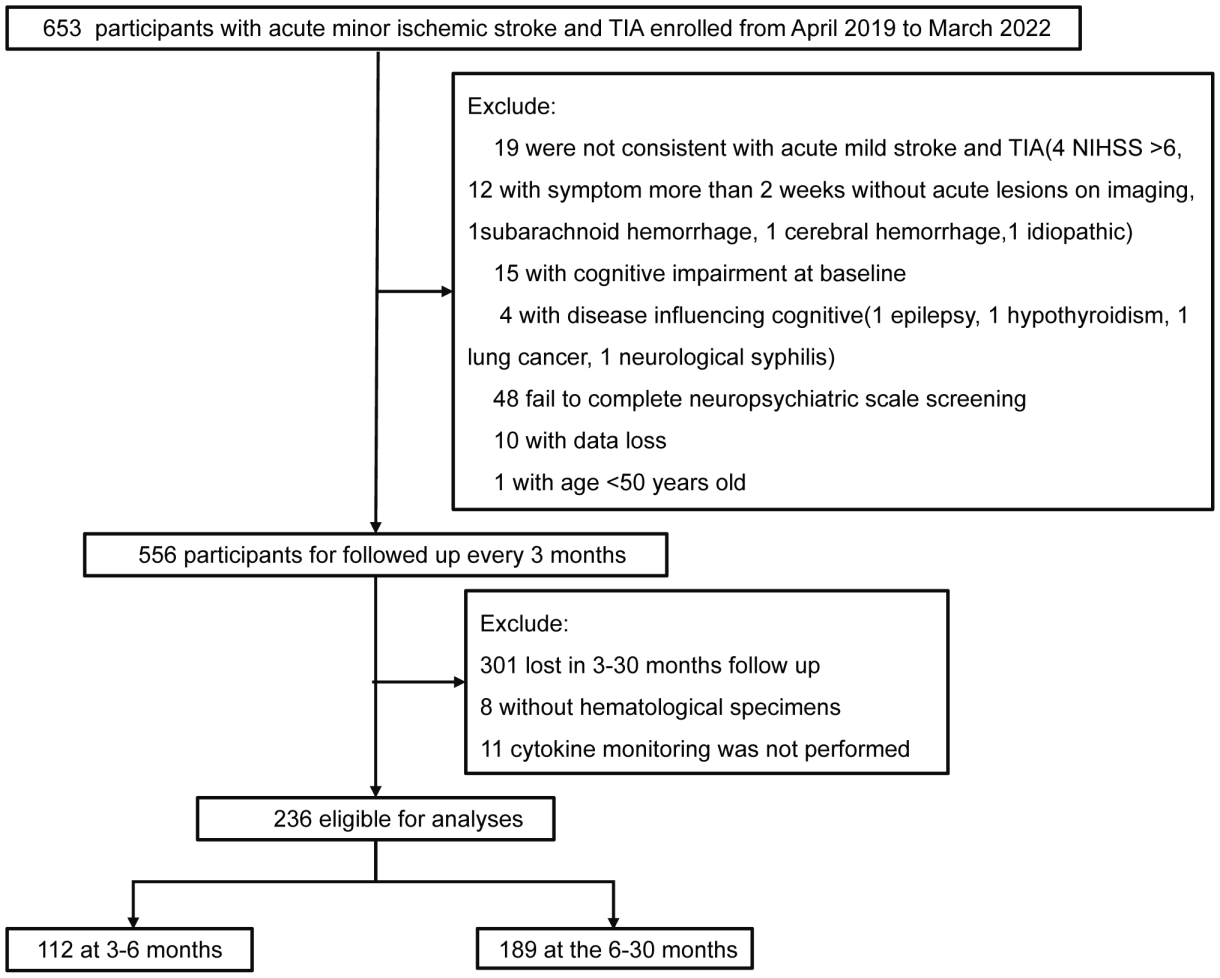
Flowchart of participant selection.

At baseline, of 236 patients, 123 had PSCI, with a prevalence rate of 52.12%. During the follow-up period of 3–30 months, 236 patients were assessed, with a median follow-up duration of 11 months (IQR: 6–12 months) and a PSCI prevalence rate of 41.53% (98 cases). A total of 112 patients were followed up within 3–6 months, with a median follow-up time of 6 months (IQR: 4–6 months) and a PSCI prevalence rate of 38.39% (43 cases). A total of 189 patients were followed up after 6 months, with a median follow-up time of 12 months (IQR: 11–14 months) and a PSCI prevalence rate of 41.27% (78 cases). Across all follow-up periods, the majority of patients were male (72%), and the median age was 62 years (**Figure 1**, **Table 1**).

**Table 1:** Baseline characteristics of patients with different follow-up duration.

Variable	Baseline	3- to 6-month follow-up	7- to 30-month follow-up	3- to 30-month follow-up
	*n* =236	*n* = 112	*N* = 189	*n* = 236
blood collection time, *d*	4 (2–6)	3 (2–6)	4 (2–5)	4 (2–6)
follow-up time, *m*		6 (4–6)	12 (11–14)	11 (6–12)
PSNCI, n(%)	113 (47.88)	69 (61.61)	111 (58.73)	138 (58.47)
PSCI, n(%)	123 (52.12)	43 (38.39)	78 (41.27)	98 (41.53)
Sex				
Male, n(%)	172 (72.88)	81 (72.32)	139 (73.54)	172 (72.88)
Female, n(%)	64 (27.12)	31 (27.68)	50 (26.46)	64 (27.12)
Age, *y*	62 (56–68)	62.5 (57–69)	62 (56–69)	62 (56–68)
Education, *y*	12 (9–15)	12 (9–12)	12 (9–15)	12 (9–15)
Atrophy, n(%)	54 (22.88)	23 (20.54)	47 (24.87)	54 (22.88)
Hypertention, n(%)	189 (80.08)	90 (80.36)	152 (80.42)	189 (80.08)
Previous stroke, n(%)	58 (24.58)	32 (28.57)	40 (21.16)	58 (24.58)
NIHSS	2 (1–3)	2 (1–3)	2 (1–3)	2 (1–3)
DWMH	1 (0–1)	1 (1–1)	1 (0–1)	1 (0–1)
DMTS, mm	11.78 (7.54–15.69)	11.35 (7.438–16.032)	12.2 (7.51–16.27)	11.78 (7.54–15.69)
NIAS	0 (0–1)	0 (0–1)	0 (0–1)	0 (0–1)
Neutrophils, 10^9^/L	4.33 (3.33–5.57)	4.27 (3.33–5.37)	3.33 (4.32–5.51)	4.33 (3.33–5.57)
TNF-α, pg/ml	7 (5.7–9.03)	7 (5.87–8.735)	6.99 (5.6–8.9)	7 (5.7–9.03)
MMP-9, pg/ml	11456 (7266–20742)	11774 (7440–22182)	11333 (7363–20193)	11456 (7266–20742)
BDNF, pg/ml	2132 (1663–2722)	2199 (1759.5–2706)	2094 (1531–2707)	2132 (1663–2722)
IL-8, pg/ml	7.1 (4.223–12.04)	7.42 (4.195–12.81)	7.1 (4.23–11.63)	7.1 (4.223–12.04)
MCP-1, pg/ml	314.56 (252.73–388.49)	308.3 (245.7–388.8)	312.6 (255.3–384.13)	314.56 (252.73–388.49)
MIP-1α, pg/ml	254.71 (161.33–370.16)	254.71 (164.32–370.16)	244.5 (155.59–358)	254.71 (161.33–370.16)
ICAM-1, pg/ml	450486.5 (319103.8–749143.5)	426858.5 (308208.2–686066.2)	447093 (302912–747848)	450486.5 (319103.8–749143.5)
IL-18, pg/ml	184.21 (130.79–267.1)	178.92 (136.19–245.85)	185.79 (130.79–275.12)	184.21 (130.79–267.1)
MPO, ng/ml	54787 (40985–75988)	52255 (38456–75567)	54873 (39963–75646)	54787 (40985–75988)
HMGB1, pg/ml	13267 (8402–20052)	13838 (9350–21334)	13090 (8535–19387)	13267 (8402–20052)

Data are presented as median and interquartile range for measurement variables and as number (%) for categorical variables. BDNF, brain-derived neurotrophic factor; DMTS, diame- ter of maximum transverse section; DWMH, deep white matter hyperintensities; HMGB1, high mobility group box1; ICAM-1, intercellular adhesion molecule-1; IL-8, interleukin-8; IL-18, interleukin-18; MCP-1, monocyte chemoattractant protein-1; MIP-1α, macrophage inflammatory protein-1α; MMP-9, matrix metalloproteinase-9; MPO, myeloperoxidase; NIAS, number of intra- cranial arterial stenosis; NIHSS, National Institutes of Health Stroke Scale; PSCI, post-stroke cog- nitive impairment; PSNCI, post-stroke no cognitive impairment; TNF-α, tumor necrosis factor-α.

### 3.2 Plasma inflammatory cytokine and PSCI

After adjusting for age, sex, education, hypertension, previous stroke, NIHSS score, DWMH, DMTS, number of acute infarcts, number of intracranial arterial stenosis, blood sample collection time, and follow-up duration, we found that, during the 3- to 30-month follow-up period, each SD increase in IL-18 was associated with a 50% increased risk of PSCI (odds ratio [OR], 1.5; 95% confidence interval [CI], 1.04–2.16; *p* = 0.03), each SD increase in MMP-9 increased the risk of PSCI by 74% (OR, 1.74; 95% CI, 1.2–2.53; *p* = 0.003), and each SD increase in MIP-1α was associated with a 49% increase in the risk of PSCI (OR, 1.49; 95% CI, 1.07–2.09; *p* = 0.021). While in dPSCI, consistent findings were observed in IL-18 and MMP-9, each SD increment with the increased risk of dPSCI by 64% (OR, 1.64; 95% CI, 1.07–2.54; *p* = 0.025) and 52% (OR, 1.52; 95% CI, 1.01–2.29; *p* = 0.046). Although no relationship was found between MIP-1α and dPSCI, IL-8 was independently correlated with dPSCI; each SD increase in IL-8 was associated with a 79% increased risk of dPSCI (OR, 1.79; 95% CI, 1.09–2.94; *p* = 0.021). Despite univariate logistic regression results suggesting that IL-18 was associated with baseline, all follow-up duration, and delayed-onset PSCI, no association was found between the above 10 cytokines and baseline or early onset PSCI in the fully adjusted models (**Table 2**).

**Table 2:** Risk of PSCI at different follow-up Periods by baseline plasma inflammatory cytokine levels.

Plasma inflammatory cytokines	Baseline PSCI			Early onset PSCI			Delayed-onset PSCI			All PSCI ^a^		
	Model 1	Model 2	Model 3 ^b^	Model 1	Model 2	Model 3	Model 1	Model 2	Model 3	Model 1	Model 2	Model 3
	OR (95%CI)	OR (95%CI)	OR (95%CI)	OR (95%CI)	OR (95%CI)	OR (95%CI)	OR (95%CI)	OR (95%CI)	OR (95%CI)	OR (95%CI)	OR (95%CI)	OR (95%CI)
IL-18	1.39 (1.04–1.88)*	1.37 (1.00–1.87)*	1.40 (0.99–1.98)	1.38 (0.87–2.18)	1.33 (0.82–2.16)	1.29 (0.70–2.39)	1.55 (1.10–2.17)*	1.65 (1.13–2.42)**	1.64 (1.07–2.54)*	1.44 (1.08–1.93)*	1.49 (1.08–2.05)*	1.50 (1.04–2.16)*
TNF-α	1.14 (0.88–1.47)	1.11 (0.85–1.46)	1.07 (0.78–1.49)	1.06 (0.72–1.56)	0.98 (0.66–1.48)	0.97 (0.61–1.54)	1.28 (0.95–1.71)	1.22 (0.89–1.69)	1.2 (0.82–1.77)	1.24 (0.96–1.61)	1.23 (0.93–1.65)	1.27 (0.91–1.79)
MMP-9	1.06 (0.82–1.37)	1.05 (0.80–1.38)	0.96 (0.7–1.32)	1.30 (0.87–1.94)	1.31 (0.86–2.01)	1.36 (0.79–2.34)	1.33 (0.98–1.8)	1.50 (1.06–2.12)*	1.52 (1.01–2.29)*	1.44 (1.09–1.91)*	1.55 (1.14–2.11)**	1.74 (1.20–2.53)**
BDNF	0.88 (0.68–1.15)	0.86 (0.64–1.15)	0.79 (0.56–1.11)	0.93 (0.66–1.31)	0.91 (0.61–1.35)	0.77 (0.45–1.34)	0.84 (0.57–1.22)	0.91 (0.60–1.37)	0.82 (0.51–1.31)	0.92 (0.7–1.21)	0.92 (0.68–1.23)	0.90 (0.62–1.30)
IL-8	0.95 (0.73–1.22)	0.93 (0.71–1.22)	0.9 (0.66–1.23)	1.04 (0.74–1.46)	0.96 (0.66–1.39)	0.85 (0.56–1.3)	1.35 (0.91–2.00)	1.54 (1.00–2.37)	1.79 (1.09–2.94)*	1.18 (0.91–1.54)	1.19 (0.89–1.60)	1.32 (0.93–1.87)
MCP-1	0.8 (0.58–1.09)	0.85 (0.61–1.19)	0.92 (0.67–1.28)	0.61 (0.35–1.06)	0.63 (0.34–1.16)	0.78 (0.36–1.68)	0.85 (0.61–1.2)	0.91 (0.60–1.39)	0.93 (0.59–1.46)	0.73 (0.51–1.04)	0.79 (0.53–1.17)	0.84 (0.54–1.29)
MIP-1α	1.08 (0.83–1.39)	1.03 (0.79–1.35)	1.09 (0.8–1.48)	1.30 (0.87–1.94)	1.22 (0.8–1.87)	1.41 (0.78–2.53)	1.21 (0.90–1.62)	1.2 (0.87–1.66)	1.39 (0.96–2.01)	1.26 (0.97–1.64)	1.24 (0.94–1.65)	1.49 (1.07–2.09)*
ICAM-1	1.07 (0.83–1.39)	1.08 (0.82–1.4)	1.07 (0.79–1.46)	1.10 (0.76–1.60)	1.1 (0.74–1.64)	1.00 (0.60–1.65)	0.97 (0.73–1.3)	0.95 (0.69–1.3)	0.91 (0.63–1.31)	1.01 (0.78–1.31)	1.02 (0.77–1.34)	1.01 (0.73–1.41)
MPO	1.02 (0.78–1.32)	0.99 (0.75–1.30)	0.87 (0.62–1.22)	1.02 (0.68–1.52)	0.98 (0.64–1.50)	1.01 (0.62–1.64)	1.19 (0.89–1.58)	1.23 (0.89–1.69)	1.19 (0.81–1.77)	1.28 (0.96–1.69)	1.3 (0.95–1.77)	1.39 (0.94–2.06)
HMGB1	1.14 (0.87–1.50)	1.16 (0.86–1.57)	1.17 (0.83–1.65)	1.12 (0.78–1.61)	1.24 (0.82–1.87)	1.37 (0.84–2.25)	0.91 (0.68–1.22)	1.94 (0.68–1.32)	0.89 (0.62–1.28)	1.00 (0.77–1.30)	1.07 (0.79–1.45)	1.03 (0.74–1.44)

a “All PSCI” refers to PSCI assessed during the 3- to 30-month follow-up period;

b baseline data without follow-up time. Model 1 unadjusted. Model 2 is adjusted for age, sex, and education. Model 3 is adjusted for age, sex, education, hypertension, previous stroke, NIHSS, DWMH, DMTS, number of intracranial arterial stenosis, brain atrophy, blood collection time, and follow-up time (*p < 0.05, **p < 0.01). BDNF, brain-derived neurotrophic factor; HMGB1, high mobility group box1; ICAM-1, intercellular adhesion molecule-1; IL-8, interleukin-8; IL-18, interleukin-18; MCP-1, monocyte chemoattractant protein-1, MIP-1α, macrophage inflammatory protein-1α; MMP-9, matrix metalloproteinase-9; MPO, myeloperoxidase; TNF-α, tumor necrosis factor-α.

**Figure 2:**
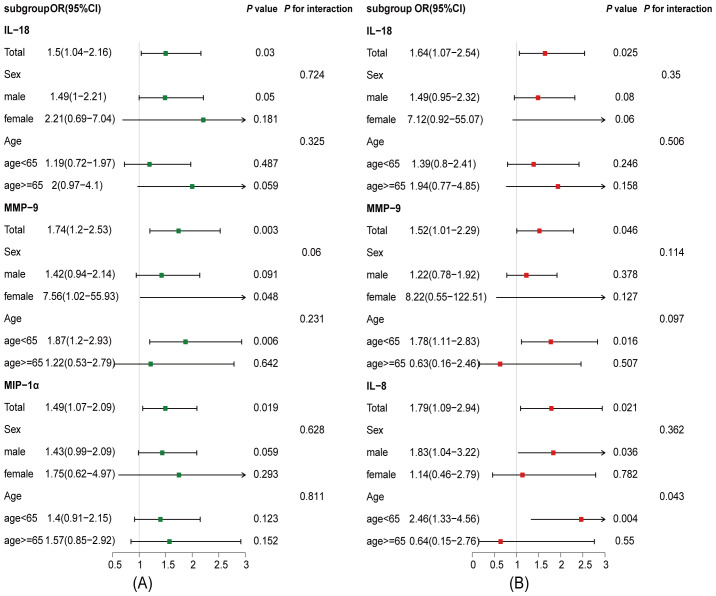
Associations between plasma inflammatory cytokines and PSCI across age and sex subgroups. The analysis was adjusted for potential confounding factors, including age, sex, education, hypertension, previous stroke, NIHSS score, DWMH, DMTS, number of intracranial arterial stenosis, brain atrophy, blood collection time, and follow-up duration. **(A)** Associations between plasma inflammatory cytokines and PSCI in age and sex subgroups at 3- to 30-month follow-up. **(B)** Associations between plasma inflammatory cytokines and delayed-onset PSCI in age and sex subgroups at 7- to 30-month follow-up. CI, confidence interval; IL-8, interleukin-8; IL-18, interleukin-18; MIP-1α, macrophage inflammatory protein-1α, MMP-9, matrix metalloproteinase-9; OR, odds ratio.

Excluding the patients with ischemic symptom onset beyond 1 week (**Supplementary Table S1**) and further excluding those with TIA (**Supplementary Table S2**), similar findings were observed. Subgroup analyses showed that the associations of IL-18, MMP-9, and MIP-1α with PSCI were consistent across age (*p* for interaction = 0.325, 0.231, and 0.811, respectively) and sex groups (*p* for interaction = 0.724, 0.06, and 0.628, respectively) (**Figure 2A**). In the dPSCI subgroup, IL-18 and MMP-9 remained consistent across age (*p* for interaction = 0.506 and 0.097, respectively) and sex strata (*p* for interaction = 0.35 and 0.114, respectively), whereas IL-8 was predominantly associated with dPSCI among patients younger than 65 years (*p* for interaction = 0.043) (**Figure 2B**).

### 3.3 Establishment and evaluation of SEM

A previous study based on the UK Biobank suggested that an increase in neutrophil levels increased the risk of vascular dementia (17). Both animal and human studies have shown that neutrophil levels increase immediately during the early stage of stroke (10, 18) and that neutrophils release MMP-9, which plays a key role in increasing the blood–brain barrier (BBB) permeability and promoting the invasion of peripheral immune cells into the injured tissue (18). Combined with the findings of this study, we selected the following variables for inclusion: peripheral inflammation biomarkers, including neutrophils, MMP-9, and IL-18; clinical data, including NIHSS score, previous stroke, and hypertension; neuroimaging variables, including DMTS, DWMH score, brain atrophy, and number of intracranial artery stenoses; demographic variables, including age, sex, and years of education; and cognitive outcome variables, including baseline and 3- to 30-month follow-up MoCA scores, with baseline and follow-up PSCI defined as MoCA scores below 22.

**Figure 3:**
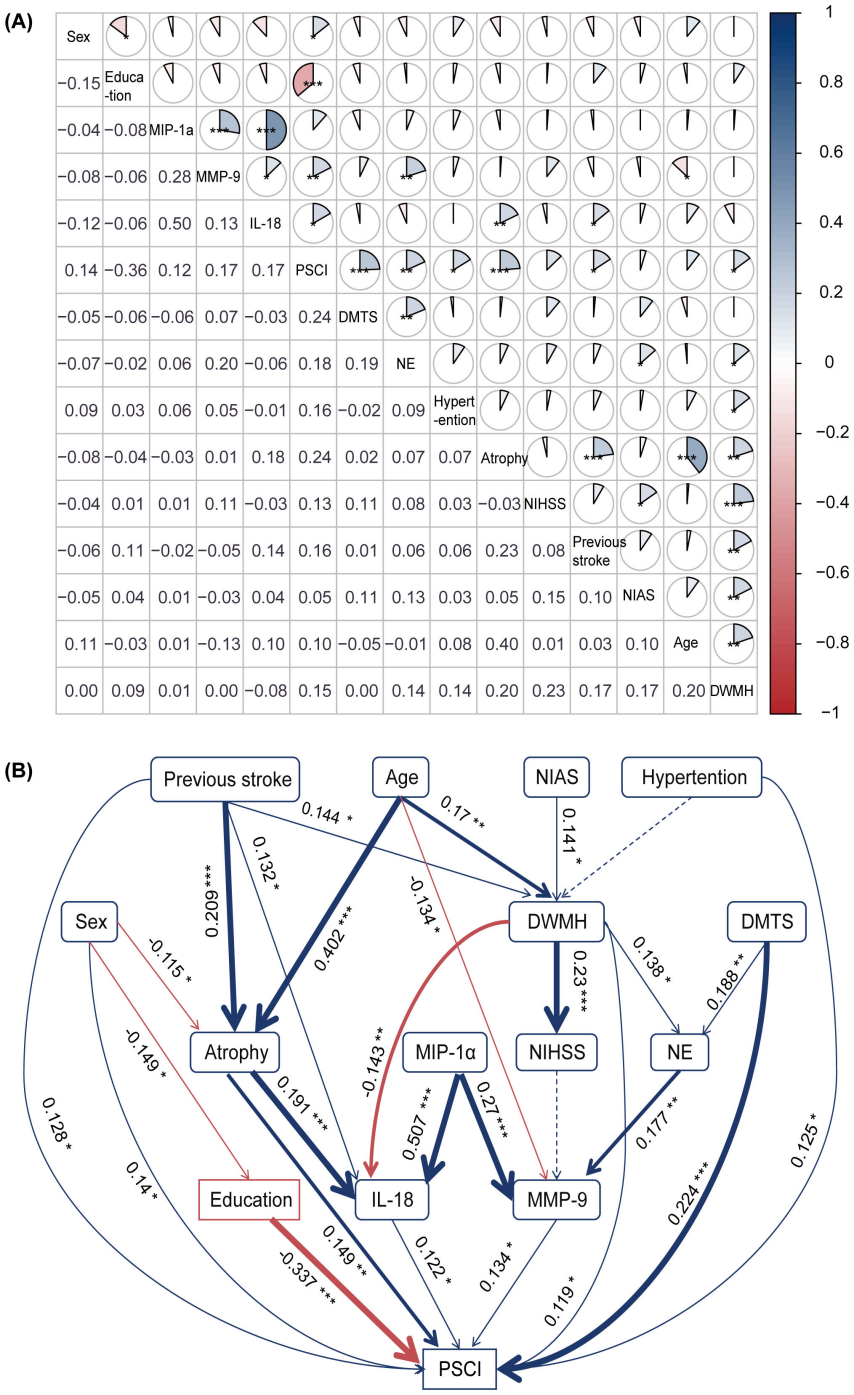
Correlation among different risk factors and the structural equation model. **(A)** The correlation analysis between various risk factors and PSCI revealed significant associations with peripheral inflammation biomarkers (e.g., neutrophils, MMP-9, and IL-18), imaging markers (e.g., DMTS, DWMH, and brain atrophy), and clinical outcomes. For instance, education level was negatively correlated with PSCI (*r* = −0.36), whereas neutrophil count, MMP-9, and IL-18 levels were positively correlated (*r* = 0.18, 0.17, and 0.17, respectively). A color gradient indicates the strength of the correlation: blue represents positive correlations, and red represents negative correlations (*p < 0.05, **p < 0.01, ***p < 0.001). **(B)** The structural equation model. Blue and red arrows indicate positive and negative relationships, respectively. Solid lines indicate significant (*p* < 0.05), while dashed lines indicate non-significant relationships. Numbers near the pathway arrows represent standardized path coefficients (*p < 0.05, **p < 0.01, ***p < 0.001). DMTS, diameter of maximum transverse section; DWMH, deep white matter hyperintensities; IL-18, interleukin-18; MIP-1α, macrophage inflammatory protein-1α; MMP-9, matrix metalloproteinase-9; NE, neutrophil; NIAS, number of intracranial arterial stenosis; NIHSS, National Institutes of Health Stroke Scale; PSCI, post-stroke cognitive impairment.

The correlations between different variables and PSCI are shown in **Figure 3A**. The SEM had a good fit with the sample (χ² = 45.284, *p* = 0.846, GFI = 0.957, CFI = 1, RMR = 0.028, SRMR = 0.037, RMSEA = 0), and it suggested both direct and indirect relationships among variables and PSCI (**Figure 3B** and **Supplementary Table S3**). The relationships among cytokines are complicated (**Supplementary Figure S1**). We further used SEM to simultaneously explore the relationships among cytokines, and the relevant SEM fitted the data well (χ² = 34.402, *p* = 0.125, GFI = 0.973, CFI = 0.989, RMR = 0.039, SRMR = 0.040, RMSEA = 0.037) (**Supplementary Figure S2**, **Supplementary Table 4**).

## 4 Discussion

This study suggests that IL-18, MMP-9, and MIP-1α are independently associated with PSCI during the 3- to 30-month follow-up period, while IL-18, MMP-9, and IL-8 are independently associated with dPSCI during the 7- to 30-month follow-up. Excluding patients with ischemic symptoms lasting more than 1 week and further excluding those with TIA did not significantly alter the observed associations. The associations between IL-18, MMP-9, and MIP-1α and PSCI remained consistent across age and sex subgroups. In cases of dPSCI, IL-8 was primarily associated with patients younger than 65 years, whereas the associations of IL-18 and MMP-9 were not influenced by age or sex. Furthermore, SEM revealed interactions among peripheral inflammatory biomarkers and suggested that MMP-9 and IL-18 may mediate the development of PSCI.

Consistent with our study findings, higher baseline blood levels of the chemokine IL-8 and MIP-1α are negatively associated with MoCA scores at 36 months after stroke (9). MMP-9 in extracellular vesicles was significantly elevated within 133 min after stroke (19), and baseline serum MMP-9 levels were associated with cognitive impairment at 3 months after stroke (20). Animal experiments indicated that IL-18 levels were higher in the hippocampus and cortex of the PSCI model induced by 45 min of middle cerebral artery embolization and reperfusion, compared with the sham group (21). Furthermore, a German study on the relationship between cognition and neuroinflammation suggested that high levels of IL-18 in the cerebrospinal fluid are associated with poorer cognitive function at baseline (22). However, no correlation between cognitive dysfunction and IL-18 was found in patients with stroke at 1- to 3-month follow-up (23). However, the above studies did not investigate correlations between IL-18, MMP-9, and PSCI across different follow-up periods. Previous studies have shown that inflammatory biomarkers measured at baseline had the strongest relationship with PSCI (9, 24). Our study evaluated inflammatory indicators at baseline and assessed patients with cognitive impairment after minor stroke and TIA at different follow-up periods, providing new insights into the etiology and treatment of PSCI.

To elucidate the different direct and indirect roles played by potential associated risk factors and cytokines in PSCI, we conducted further SEM analysis based on the above findings and previous correlation research. We found that IL-18, MMP-9, DMTS, DWMH, brain atrophy, previous stroke, hypertension, and female sex were directly and positively correlated with PSCI. The release of IL-18 was primarily associated with brain load (brain atrophy, DWMH, and previous stroke), while NE–MMP-9 appeared to be more closely related to acute cerebral ischemia (DMTS). Consistent with our study findings, a recent study suggested that increased cerebrospinal fluid IL-18 levels at baseline were associated with longitudinal decreases in gray matter volume in the hippocampus, thalamus, and basal ganglia and in white matter volume in the frontal area (22). A previous U.S. cohort study demonstrated that an inflammatory network centered on serum IL-18 levels at baseline was associated with white matter lesions within 6 months after baseline (25). However, our study suggested a negative correlation between the severity of DWMH at stroke onset and IL-18 levels. Further large-sample clinical studies are needed to explore the relationship between IL-18 and white matter lesions at different times after stroke. Regarding MMP-9, previous studies have shown that it is associated with infarct volume (26) but not with stroke severity (27), consistent with our findings. It is well-established that education level is a protective factor for PSCI and that women are generally less educated than men. In addition, studies have suggested that women suffer from more severe strokes, which may explain why they are more likely to develop PSCI (28), as observed in our study.

Cytokine interactions are highly complex, and the upstream and downstream signaling pathways remain poorly understood. Following an acute stroke or TIA, microglia—the resident immune cells of the central nervous system—are rapidly activated, initiating sterile inflammation, promoting infiltration of peripheral immune cells, and releasing inflammatory mediators that regulate tissue damage and repair (29). HMGB1 levels are significantly elevated in activated microglia, and extracellular HMGB1 may enhance the production of TNF-α and IL-18 via Toll-like receptors, NF-κB, and related pathways (30). As a pro-inflammatory factor, IL-18 promotes microglial polarization toward the M1 phenotype, thereby amplifying the inflammatory response (31). The absent in melanoma 2 (AIM2) inflammasome is upregulated in M1-polarized microglia and facilitates IL-18 maturation and secretion via caspase-1 activation (32). Additionally, IL-18 activates NK cells, type I innate lymphoid cells, and Th1 cells through the NF-κB and MAPK pathways, thereby promoting type I immunity (33); it also induces TNF-α production (34, 35) and ICAM-1 expression (36). TNF-α induces glutamate release from neurons and glial cells, leading to leukocyte infiltration, tissue damage, and the subsequent production of chemokines (30), such as IL-8 and MIP-1α. IL-8 is a key chemoattractant and activator of neutrophils, promoting the release of potent cytotoxic mediators such as MMP-9, reactive oxygen species, and neutrophil extracellular traps (37). Sustained neutrophil activation likely contributes to continued vascular injury, chronic BBB disruption, and delayed parenchymal damage, thereby driving progressive cognitive decline. The age-specific association observed in patients younger than 65 years may reflect differential neutrophil responsiveness (17). The specific association of IL-8 with dPSCI, especially in younger individuals, highlights the pivotal role of neutrophil-mediated injury in the progression of cognitive impairment.

MIP-1α induces TNF-α production (38) and upregulates the expression of ICAM-1 and IL-18. As a key chemokine, MIP-1α also promotes leukocyte chemotaxis (9). Infiltrating monocytes and macrophages differentiate into pro-inflammatory subsets within the brain parenchyma, while T cells secrete cytotoxic factors. These immune cells and their associated mediators amplify local inflammation, exacerbate oxidative stress, and sustain a cycle of tissue injury and impaired repair (39), contributing to cognitive decline over the 3–30-month period. MIP-1α and IL-8 enhance endothelial adhesion via ICAM-1 and stimulate the release of MMP-9 and MPO. MMP-9, secreted by activated microglia, infiltrating leukocytes, and endothelial cells in response to cytokines such as TNF-α and IL-18, degrades the extracellular matrix and tight junctions, thereby increasing the BBB permeability (26) and promoting infiltration of peripheral immune cells. Secondary oxidative stress leads to excessive production of reactive oxygen species, which in turn promotes the release of pro-inflammatory cytokines such as IL-18 and TNF-α (40). Furthermore, MMP-9 can directly damage neurons and synapses, impair angiogenesis, and potentially influence amyloid-β pathways, resulting in a multifaceted assault on cognitive networks (41). This initiates a vicious cycle of inflammation, amplifying neuroinflammation and parenchymal injury, and ultimately contributing to neuronal loss and synaptic dysfunction underlying cognitive impairment.

These pathways collectively initiate a cascade of inflammatory responses, including cytokine release, chemokine induction, and enhanced adhesion and migration of leukocytes and macrophages to the injured brain tissue (36). These processes contribute to secondary brain injury, BBB disruption, demyelination, impaired angiogenesis, and reduced synaptic plasticity, ultimately promoting lymphocyte infiltration in the late phase of stroke and contributing to the development of PSCI (30, 42). Our findings demonstrate that IL-18 and MMP-9 are consistently associated with cognitive impairment across both the 3- to 30-month (PSCI) and 7- to 30-month (dPSCI) follow-up periods, MIP-1α is associated with cognitive impairment during the 3- to 30-month period, and IL-8 during the 7- to 30-month period. These results suggest that these post-stroke neuroinflammatory mediators actively contribute to the chronic processes underlying cognitive decline, in alignment with the core inflammatory cascade described above. However, microglia display a spectrum of phenotypes that vary depending on disease stage and context. The classical M1/M2 polarization framework is increasingly regarded as overly simplistic, as distinct microglial phenotypes can coexist and interconvert, suggesting a continuum of activation states (43). The specific phenotypic shifts involved in PSCI pathogenesis remain to be elucidated.

A previous meta-analysis has suggested that anti-inflammatory interventions, such as complement inhibition and fingolimod administered within 24h after stroke, are promising therapeutic approaches to alleviate PSCI in animal models (8). Furthermore, animal models of middle cerebral artery embolization and reperfusion have suggested that activation of glial and endothelial cells produces AIM2 inflammasomes, which stimulate increased production of the downstream cytokines IL-1β and IL-18, possibly exacerbating PSCI. AIM2 knockout mice showed significant improvement in cognitive function, suggesting that the AIM2 inflammatory pathway may be a potential target for therapy (21). At the same time, IL-8 and MCP-1 can promote the release of the neurotrophic factor BDNF. Additionally, a multicenter study on patients with hypertension and ischemic stroke suggested that high serum BDNF levels were associated with a decreased incidence of PSCI at 3 months (44). However, our study did not find a direct relationship between BDNF and PSCI, possibly because the included patients had conditions beyond hypertension alone. The above pathways provide some potential mechanisms underlying the inflammatory pathways of PSCI, which require further investigation in animal experiments and multi-center clinical trials.

In this study, we aimed to elucidate the interactions between traditional risk factors, peripheral inflammatory factors, and their interactions with PSCI using SEM, which provides a novel perspective and methodological approach for exploring disease pathogenesis. However, our study had some limitations. First, SEM can illustrate only correlation, not causation. The construction of SEM is limited by the sample size and restricted inclusion of influencing factors. Second, although MMSE and MoCA are widely used to evaluate PSCI, they may lack sensitivity and specificity for detecting mild to moderate cognitive impairment (45). The patient’s loss occurred between the early- and delayed-onset stages, limiting the objective assessment of the impact of cytokines during this stage. Third, various inflammatory factors affect cognitive function differently at different stages after stroke. Although we performed some longitudinal assessments using cognitive scales, we did not measure cytokine levels repeatedly. Finally, although the statistical analysis revealed a weak correlation between the inflammatory factors IL-18 and MMP-9 and the outcome variable, with standardized path/regression coefficients of 0.122 and 0.134, respectively, other well-recognized indicators, such as brain atrophy, white matter hyperintensity, and previous stroke, showed coefficients of 0.149, 0.119, and 0.128, respectively. This may be due to the complex etiology and pathogenesis of PSCI. Furthermore, inflammation is a complex process, and developing more accurate treatment options will require additional, multi-center, large-scale clinical studies and relevant animal experiments.

## 5 Conclusions

IL-18 and MMP-9 were independently associated with PSCI at both the 3- to 30-month and 7- to 30-month follow-ups. MIP-1α was independently associated with PSCI at the 3- to 30-month follow-up, while IL-8 was associated with PSCI at the 7- to 30-month follow-up. Long-term cognitive impairment after mild stroke and TIA results from the interaction between the ischemic severity and the patient’s pre-existing prior brain burden. Inflammation-mediated mechanisms centered on IL-18 and neutrophils–MMP-9 interactions are involved in the development of PSCI, paving potential avenues for research into inflammatory pathways in PSCI.

## Data Availability

The raw data supporting the conclusions of this article will be made available by the authors, without undue reservation.
