# Supplementary material for: Plasma inflammatory cytokines are associated with cognitive impairment after acute minor ischemic stroke and transient ischemic attack
Source: Front Immunol. 2025 Aug 1;16:1445938. doi: 10.3389/fimmu.2025.1445938 (PMC12353376; doi:10.3389/fimmu.2025.1445938)
Supplement: Supplementary file 1 [file DataSheet1.docx]

Supplementary Material

Article TitlePlasma Inflammatory Cytokines are Associated with Cognitive Impairment after Acute Minor Ischemic Stroke and Transient Ischemic Attack

**PanPan Zhao^1^, Meng Zhao^1^, GuiMei Zhang^1^, WeiJie Zhai^1^, YongChun Wang^1^, YanXin Shen^1^, Li Sun^1^***

**^1^Department of Neurology and Neuroscience Center, The First Hospital of Jilin University, Jilin University, Changchun, China**

*** Correspondence:** Li Sun: sunli99@jlu.edu.cn

# Supplementary Figures and Tables

## Supplementary Figures

## **Supplementary Figure 1.** correction between cytokines.

##
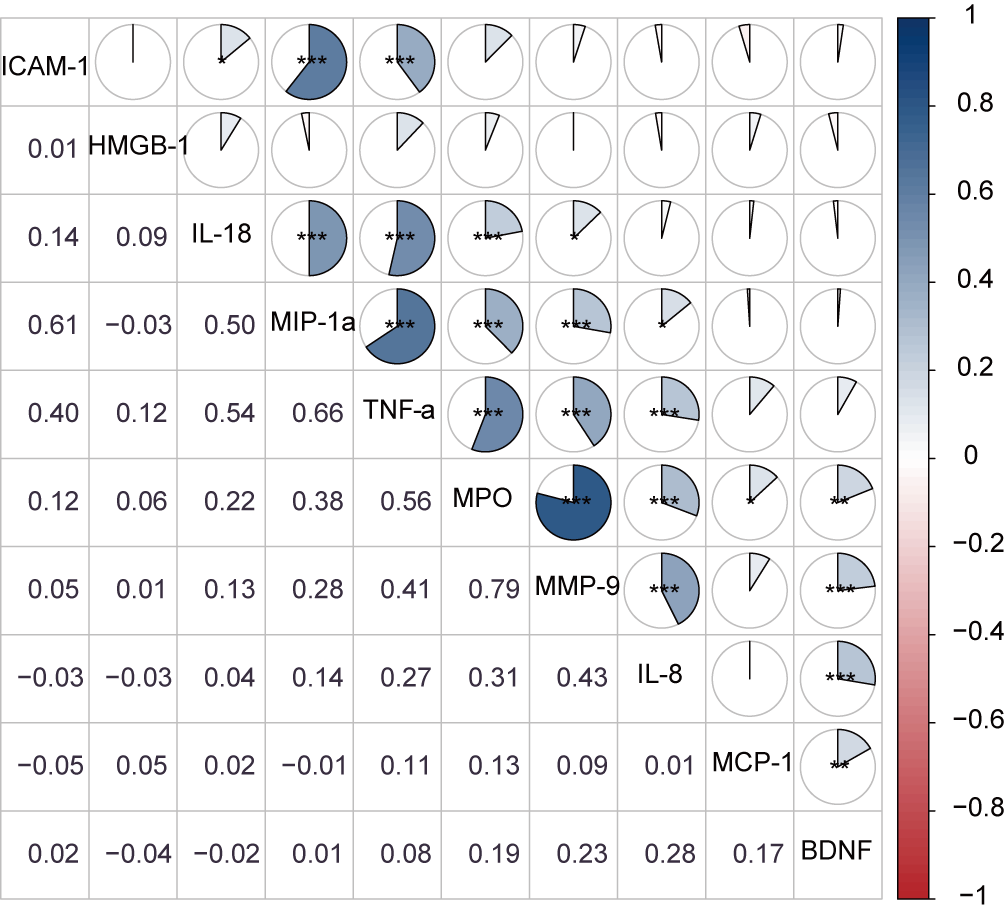


## (**p* < 0.05, ***p* < 0.01,****p* < 0.001). Abbreviations: BDNF = brain-derived neurotrophic factor, HMGB 1 = high mobility group box 1, ICAM-1 = intercellular adhesion molecule-1 , IL-8 = interleukin-8, IL-18 = interleukin-18, MCP-1 = monocyte chemoattractant protein-1, MIP-1α= macrophage inflammatory protein-1α, MMP-9 = matrix metalloproteinase-9, MPO = myeloperoxidase, TNF-α= tumor necrosis factor-α.

## **Supplementary Figure 2.** The structural equations model on cytokines.

##
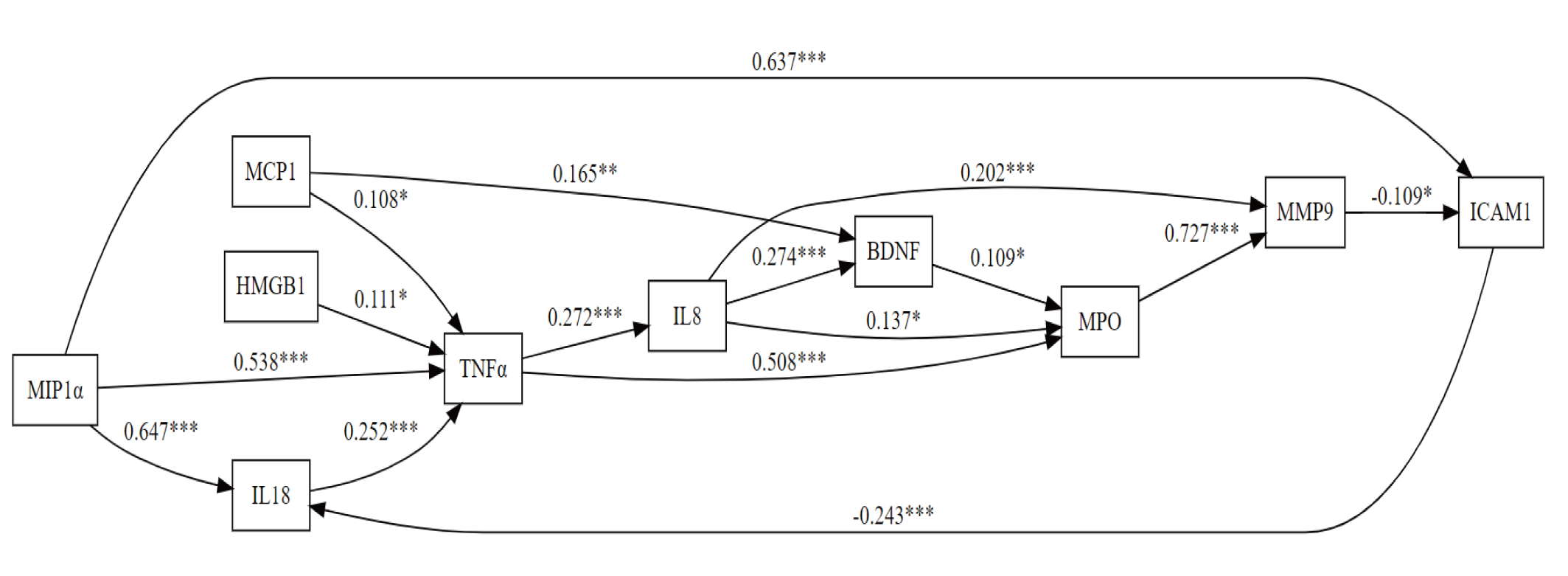


Numbers near the pathway arrow indicate the standard path coefficients.(**p* < 0.05, ***p* < 0.01,****p* < 0.001). Abbreviations: BDNF = brain-derived neurotrophic factor, HMGB1 = high mobility group box 1, ICAM1 = intercellular adhesion molecule-1, IL8 = interleukin-8, IL18 = interleukin-18, MCP1 = monocyte chemoattractant protein-1, MIP1α = macrophage inflammatory protein-1α, MMP9 = matrix metalloproteinase-9, MPO = myeloperoxidase, TNFα = tumor necrosis factor-α.

## Supplementary Tables

**Supplementary Table 1.** Risk of PSCI at different follow-up Periods by baseline plasma inflammatory cytokine levels (excluding blood collection time > 7 days)

a “All PSCI” refers to PSCI assessed during the 3–30-month follow-up period; b baseline data without follow-up time. Model 1 unadjusted. Model 2 is adjusted for age, sex, and education. Model 3 is adjusted for age, sex, education, hypertension, previous stroke, NIHSS, DWMH, DMTS, number of intracranial arterial stenosis, brain atrophy, blood collection time, and follow-up time. (**p* < 0.05, ***p* < 0.01). Abbreviations: BDNF = brain-derived neurotrophic factor, HMGB1 = high mobility group box 1, ICAM-1 = intercellular adhesion molecule-1, IL-8 = interleukin-8, IL-18 = interleukin-18, MCP-1 = monocyte chemoattractant protein-1, MIP-1α = macrophage inflammatory protein-1α, MMP-9 = matrix metalloproteinase-9, MPO = myeloperoxidase, TNF-α = tumor necrosis factor-α.

**Supplementary Table 2.**  Risk of PSCI at different follow-up Periods by baseline plasma inflammatory cytokine levels (excluding patients with TIA and blood collection time > 7 days)

a “All PSCI” refers to PSCI assessed during the 3–30-month follow-up period; b baseline data without follow-up time. Model 1 unadjusted. Model 2 is adjusted for age, sex, and education. Model 3 is adjusted for age, sex, education, hypertension, previous stroke, NIHSS, DWMH, DMTS, number of intracranial arterial stenosis, brain atrophy, blood collection time, and follow-up time. (**p* < 0.05, ***p* < 0.01). Abbreviations: BDNF = brain-derived neurotrophic factor, HMGB1 = high mobility group box 1, ICAM-1 = intercellular adhesion molecule-1, IL-8 = interleukin-8, IL-18 = interleukin-18, MCP-1 = monocyte chemoattractant protein-1, MIP-1α = macrophage inflammatory protein-1α, MMP-9 = matrix metalloproteinase-9, MPO = myeloperoxidase, TNF-α = tumor necrosis factor-α.

**Supplementary Table 3.** Regression statistics in structural equations model on risk factors

Abbreviations: DMTS = diameter of maximum transverse section, DWMH = deep white matter hyperintensities, IL-18 = interleukin-18, MIP-1α = macrophage inflammatory protein-1α, MMP-9 = matrix metalloproteinase-9, NIAS = number of intracranial arterial stenosis, NIHSS = National Institutes of Health Stroke Scale, PSCI = post-stroke cognitive impairment.

**Supplementary Table 4.**  Regression statistics in structural equations model on cytokines

Abbreviations: BDNF = brain-derived neurotrophic factor, HMGB1 = high mobility group box 1, ICAM-1 = intercellular adhesion molecule-1, IL-8 = interleukin-8, IL-18 = interleukin-18, MCP-1 = monocyte chemoattractant protein-1, MIP-1α = macrophage inflammatory protein-1α, MMP-9 = matrix metalloproteinase-9, MPO = myeloperoxidase, TNF-α = tumor necrosis factor-α.
